# Technical Refinements in Single-Port Laparoscopic Surgery of Inguinal Hernia in Infants and Children

**DOI:** 10.1155/2010/392847

**Published:** 2010-05-23

**Authors:** Yu-Tang Chang

**Affiliations:** ^1^Division of Pediatric Surgery, Department of Surgery, Kaohsiung Medical University Hospital, Kaohsiung Medical University, 100 Tzyou 1st Road, Kaohsiung 80708, Taiwan; ^2^Graduate Institute of Medicine, College of Medicine, Kaohsiung Medical University, Kaohsiung 80708, Taiwan; ^3^Department of Surgery, Faculty of Medical School, College of Medicine, Kaohsiung Medical University, Kaohsiung 80708, Taiwan

## Abstract

The techniques of minimal access surgery for pediatric inguinal hernia are numerous and they continue to evolve, with a trend toward increasing use of extracorporeal knotting and decreasing use of working ports and endoscopic instruments. Single-port endoscopic-assisted percutaneous extraperitoneal closure seems to be the ultimate attainment, and numerous techniques have mushroomed in the past decade. This article comprehensively reviews and compares the various single-port techniques. These techniques mainly vary in their approaches to the hernia defect with different devices, which are designed to pass a suture to enclose the orifice of the defect. However, most of these emerging techniques fail to entirely enclose the hernia defect and have the potential to lead to higher incidence of hernia recurrence. Accompanying preperitoneal hydrodissection and keeping identical subcutaneous path for introducing and withdrawing the suture, the suture could tautly enclose the hernia defect without upper subcutaneous tissues and a lower peritoneal gap, and a trend towards achieving a near-zero recurrence rate.

## 1. Introduction

Traditional inguinal herniotomy is a well-developed surgical technique for uncomplicated inguinal hernia in infants and children. It usually necessitates one small 1.5 to 2 cm skin incision, and the possible postoperative complications, such as recurrence or injury to the vas deferens, are not high [1]. Laparoscopic surgery has recently emerged as an alternative in its management. Although not as widely used as conventional open herniotomy, laparoscopic herniorrhaphy has clear advantages, especially those related to the evaluation of possible contralateral opening and a safe high ligation of the hernia sac at the internal ring without injury to the vas deferens and spermatic vessels [2]. In 1997, El-Gohary first described laparoscopic ligation of inguinal hernia in girls [3]. Subsequently, numerous technical reports for the laparoscopic hernia repair in children have evolved [2].

Although modifications on laparoscopic surgery continue to be refined, there are some technical limitations, which influence a pediatric surgeon's willingness to perform the procedure [2]. The universally known limitations of the laparoscopic surgery are (1) most of these methods employ a laparoscope inserted via an umbilical incision and two lateral ports for instruments to ligate the hernia defect [4]. The necessity for intraabdominal skills, such as intracorporeal suturing, knot-tying, and manipulation of the suture on a needle may be time-consuming and cumbersome [5]. (2) Recurrence rate after laparoscopic surgery is generally known to be higher than after open surgery [1, 4]. Partial omission of the defect circumference, strength and appropriateness of the knot, inclusion of tissue other than peritoneum in the suture with a propensity for subsequent loosening, use of absorbable sutures, and failure to detect a rare or direct hernia are some reported factors contributing to recurrence in laparoscopic surgery [2]. (3) Compared to open herniotomy with an almost disappeared wound in the skin crease, laparoscopic approach did not take any superiority in cosmesis [6]. Conversely, the procedure was thought not to be minimally invasive because of the necessity of multiple skin incisions and pneumoperitoneum during operation. In a single-blinded, randomized study, recovery and outcome were similar after open and three-port laparoscopic hernia repair in children. Moreover, three-port laparoscopic approach was associated with increased operative time and postoperative pain [6].

 To enhance a pediatric surgeon's willingness, further development is intended to decrease the number and size of skin incisions, lower the recurrence rate, and simplify or avoid intracorporeal technique [2]. From above conception, single-port endoscopic-assisted percutaneous extraperitoneal closure seems to be the ultimate attainment and numerous techniques have mushroomed in the past decade [5, 7–11]. Herein, the author reviews the literature in an attempt to compare the various approaches of the latest advancement in pediatric hernia surgery.

## 2. Surgical Technique

 Of single-port laparoscopic surgery for pediatric inguinal hernia, the suture was always introduced and withdrawn percutaneously at the corresponding skin of the orifice of the hernia defect by variable devices, and was tied extracorporeally to obliterate the hernia sac. The knot was then placed in the subcutaneous space. Reported single-port techniques with extracorporeal knotting are shown in Table 1 [5, 7–11].

### 2.1. Technique of Subcutaneous Endoscopically Assisted Ligation (SEAL)

The first described is Harrison et al. in 2005 with subcutaneous endoscopically-assisted ligation (SEAL) of the hernia defect [7]. The SEAL technique has been performed since 2001 [8]. Using only the camera port and passing a suture on a large swaged-on needle percutaneously to enclose the defect, knot-tying was performed extracorporeally. In 2007, the same group described the early result of 300 inguinal hernias [8]. Overall complications occurred in 15.7% of patients and a recurrence rate of 4.3% was comparable to prior series of laparoscopic repairs. However, the known limitations of the SEAL technique are (1) for successful mating and guidance, the entry point of both the needle and the track should exactly match the curve of the needle. If the curve of the needle could not conform to the configuration of the ring, it would be difficult to pass the needle through the posterior hemicircumference of the ring. The needle may jump over the vas and vessels and a peritoneal gap may be left untouched. (2) Two stab incisions are necessary for the swaged-on needle and the receiving Tuohy needle, and a depression or fold of the corresponding skin might sometimes occur if the knot-tying is not placed in the correct deeper plane [5]. (3) If the size of the defect is extraordinarily large, an additional instrument to assist guidance of the needle or conversion to open herniotomy is necessary [8, 12, 13].

In 2008, Bharathi et al. modified the technique of SEAL [5]. A small amount of saline was injected using a hypodermic or spinal needle in the retroperitoneal space (preperitoneal hydrodissection) to lift up the peritoneum of the vas and the vessels. The suture could be then advanced to encircle the posterior hemicircumference of the defect completely. If the saline injection should fail, the authors would take as much as the circumference of the defect as was possible without collateral damage by the first suture. Then, this allowed a second, separate loop to encircle the defect. However, multiple stab incisions at the corresponding skin were always necessary.

### 2.2. Technique of Percutaneous Internal Ring Suturing (PIRS)

 In 2006, Patkowski et al. described the technique of percutaneous internal ring suturing (PIRS) for inguinal hernia in children [9].An 18-gauge injection needle with a nonabsorbable suture inside the barrel of the needle was placed through the abdominal wall into the peritoneal cavity. By moving the injection needle, the suture passed under the peritoneum around the hernia defect. The knot was tightened extracorporeally and placed in the subcutaneous space. The PIRS technique required only one umbilical port and one needle puncture point. However, as in the original SEAL technique, a peritoneal gap of the suture at the location of vas and vessels was still left untouched.

### 2.3. Technique with a Vascular Catheter, a Hooked Pin, and Preperitoneal Hydrodissection

**Figure 1:**
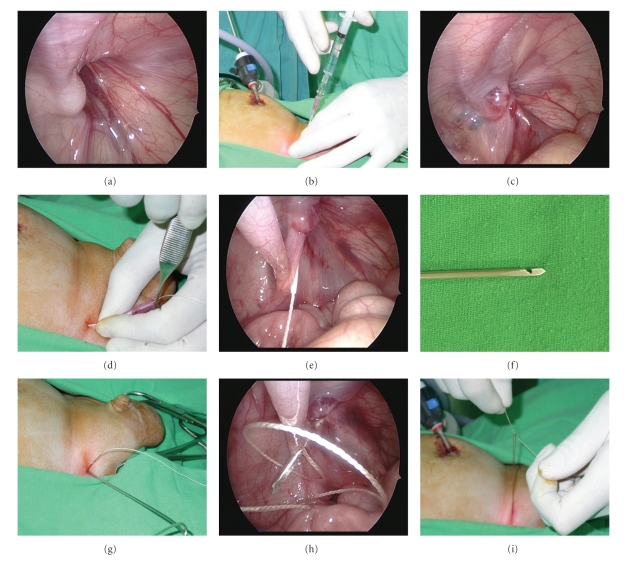
Intraoperative photo showing a 2-year-old boy receiving the hooked pin method. (a) Note right side inguinal hernia and the close proximity of the vas deferens (V) and testicular vessels (T) to the ring. (b) Introduction of the vascular catheter into the preperitoneal space along left side of the hernia defect. (c) The “preperitoneal hydrodissection” method. Injection of normal saline via the vascular catheter separates the vas and vessels from the peritoneum and allows the vascular catheter (arrow) to cross over. (d, e) The indwelling needle was removed, and a nonabsorbable suture was threaded through the sheath of the catheter, with the other end of the suture remaining above the skin. The sheath was then withdrawn. (f) The hook-pin device was easily made by modifying a pin used in orthopedic surgery. The device has a hook near the tip for catching hold of the suture. (g, h) Through the same stab incision, the hook-pin was introduced along the opposite side of the hernia defect into the intraabdominal space to pick up the silk, and the suture was then pulled through the abdominal wall. (i) The hernia defect was closed and the circuit suturing was tied extracorporeally.

**Figure 2:**
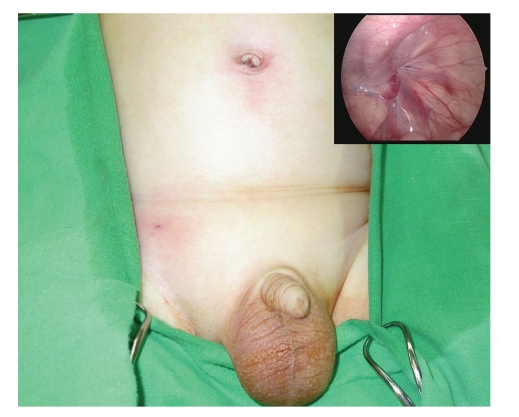
The final wound appearance of the inguinal hernia repair (arrows) and the hernia defect after the suture was tied (upper inset).

 In 2008, the author developed a modified technique of SEAL and PIRS [10]. Under the laparoscopic guidance, the hernia defect was enclosed by a nonabsorbable suture, which was introduced into the abdomen by an 18 Fr vascular catheter (Surflash I.V. catheter, I.D. 0.95 × 64 mm, Terumo Corporation, Tokyo, Japan) on one side of the hernia defect and withdrawn on the opposite side by a hooked pin, which was made by an orthopedic pin (I.D. 1.8 mm, MES-CF01-063-21, Mizuho, Tokyo, Japan), through one needle puncture wound (Figure 1). During the procedure, 5 to 8 mL of isotonic saline solution were infused via the needle into the preperitoneal space to obtain the preperitoneal dissection of the hernia defect. The author started to perform the surgical technique in March 2007. From March 2007 to January 2010, a total of 288 procedures were performed among 201 consecutive infants and children. Of the technique, only one umbilical trocar wound and another stab incision were made (Figure 2). Besides, the hernia defect could be enclosed completely without a lower peritoneal gap since preperitoneal hydrodissection could safely separate the peritoneum from the vas and the vessels. Since the used vascular catheter and hooked pin were long enough (64 mm and 300 mm, resp.), failure to lift up the peritoneum entirely was rare. However, some upper subcutaneous tissues, including nerves and muscles, may cause injury by their inclusion in the upper portion of the circuit suturing. The inclusion of unnecessary subcutaneous tissues in the ligature may lead to a propensity for subsequent loosening of the knot, causing later recurrence [2].

### 2.4. Technique with a Hooked Injection Needle and Preperitoneal Hydrodissection

Later, the author described the modification of the hooked pin method with a homemade hooked injection needle (Optiva I.V. Catheter Radiopaque, I.D. 1.8 × 50 mm, Ethicon Endo-surgery, Johnson-Johnson Company), which is designed to traverse the suture and cause hydrodissection to the preperitoneal space [11]. During the procedure, the tip of the hooked injection needle was kept beneath the fascia at the period after introducing and before pulling the suture. Thus, the suture could tautly enclose the hernia defect without upper subcutaneous tissues and a lower peritoneal gap.

## 3. Discussion

**Figure 3:**
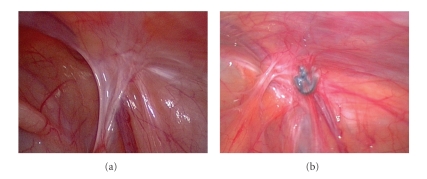
Laparoscopic views of 17 months (a) and 24 months (b) after traditional open herniotomy for right side inguinal hernias. Without intraabdominal manipulation, open herniotomy still causes local intraperitoneal adhesion (arrows) at the original entrance into the hernia sac. The peritoneal adhesions may be caused by suture ligation of the sac and subsequent tissue reaction of the sutures. V, vas deferens; T, testicular vessels

**Figure 4:**
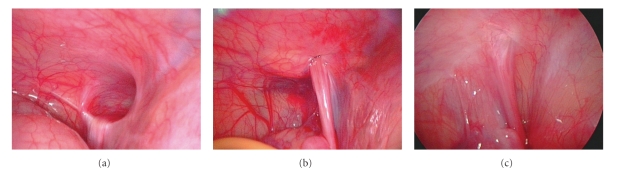
A 2-year-old girl receiving the hooked pin method. (a) Note left side inguinal hernia before operation. (b) The hernia defect was closed after operation. (c) Laparoscopic surgery for other reasons was performed 94 days after operation. Note the peritoneal scarring occurred in closure of the hernia defect.

 Postsurgical peritoneal adhesions are a consequence of injured peritoneal surface (including incision, cauterization, suturing, or other means of trauma) fusing together to form scar tissue [14]. Of the inguinal hernia sac, the endothelium is the continuity of peritoneal mesothelium. In the open herniotomy, trauma due to traverse of the suture and tissue reaction of the suture material may also cause peritoneal adhesion and fibrosis (Figure 3). Since the tensile strength of any suture may diminish eventually, the author suggests that peritoneal adhesion and fibrosis may be the leading factor for complete obliteration of the hernia defect in the long run after either open herniotomy or laparoscopic surgery (Figure 4). Thus, how can adequate peritoneal adhesions during hernia operation in the era of minimal access surgery are applied? Since partial omission of the defect circumference was the reported factor contributing to recurrence in laparoscopic surgery [2], completely enclosing the hernia defect without gaps, the same as suture ligation in the open herniotomy, is crucial to moving towards a near-zero recurrence rate.

However, in the standard three-port technique with intracorporeal knot-tying or the two-port technique with an assistant port for intraabdominal suturing, the hernia defect was always closed by N-shaped or purse-string sutures, both of which cannot enclose the defect completely and may leave multiple peritoneal gaps. The resultant peritoneal gaps cannot provide adequate peritoneal injury and may disturb or defer further peritoneal adhesion if the knot-tying is loosening gradually, leading to potential recurrence. The author suggests that complete extraperitoneal enclosing of the hernia defect could decrease peritoneal gaps, and single-port endoscopic-assisted percutaneous extraperitoneal closure may be the preferred technique. Moreover, in the single-port technique, the ligation of the hernia defect could be achieved percutaneously without the need for intracorporeal manipulation of the needle and knot-tying.

To completely enclose suture of the hernia defect without any gap in the single-port technique, preperitoneal hydrodissection must be the main step. The concept of hydrodissection during laparoscopic hernia repair has been already described in the literature [15]. In 2007, Chan et al. employed preperitoneal hydrodissection in the three-port intraperitoneal-suturing technique, and concluded that the recurrence rate could decrease from 4.88 to 0.4% after the usage of preperitoneal hydrodissection [15]. Recently, the method of preperitoneal hydrodissection has been applied in the single-port technique [5, 10, 11]. With the aid of hydrodissection, the vas and vessels could be separated from the peritoneum; therefore, a completely enclosing suture of the hernia defect could be provided without any gaps [5, 10, 11].

Meanwhile, the method of preperitoneal hydrodissection was useful in (i) providing additional space for negotiating the working instruments, (ii) keeping the device just under the peritoneum, and observing the needle sign [8], (iii) avoiding injury to the vas and vessels, (iv) making a further airtight extracorporeal knot-tying [15], and (v) decreasing postoperative hydrocele, which may be caused by interruption of testicular lymphatic drainage because of being thicker than the peritoneum bites of the encircling suture [5]. Moreover, normal saline, the solution for preperitoneal hydrodissection, could predispose the formation of peritoneal adhesions and fibrosis [16]. Therefore, during passing of the suture, preperitoneal normal saline injection may cause more tissue trauma, further promote the formation of peritoneal adhesions and minimize later recurrence (Figure 4).

However, being a technique of percutaneous closure of inguinal hernia, simultaneous ligation of subcutaneous tissues between the skin and hernia defect was inevitable [5, 7–10]. This might possibly increase the recurrence rate when subsequent loosening of the knot takes place. Accompanying preperitoneal hydrodissection and keeping identical subcutaneous path for introducing and withdrawing the suture, the latest reported single-port technique could overcome the limitations and tautly enclose the hernia defect without upper subcutaneous tissues and a lower peritoneal gap [11].

## 4. Conclusions

Preperitoneal hydrodissection could completely enclose the hernia defect without peritoneal gaps, whereas keeping identical subcutaneous path during traversing the suture could avoid simultaneous ligation of subcutaneous tissues between the skin and hernia defect. Furthermore, the smaller and fewer skin incisions of the single-port technique could reach the state of minimally invasive surgery. However, single-port laparoscopic surgery for pediatric inguinal hernia is a technique in evolution. More long-term follow-up concerning the recurrence rate is necessary.

## Figures and Tables

**Table 1:** Reported single-port technique with extracorporeal knotting.

Studies (1st author)	Technique	Port size (mm)	Number of associated stabbing incisions	Complete ring	Subcutaneous tissue inclusion	Tensionless knot tying	Protection of vas and vessels
Harrison et al. 2005 [7]	SEAL	2.7	2 (unilateral)	−, small gap	+	−	+, jump over them
			4 (bilateral)				
Ozgediz et al. 2007 [8]	SEAL	2.7	2 (unilateral)	−, small gap	+	−	+, jump over them
			4 (bilateral)				
Patkowski et al. 2006 [9]	PIRS	2.5 or 5	1 (unilateral)	−, small gap	+	−	+, jump over them
			2 (bilateral)				
Bharathi et al. 2008 [5]	Modified SEAL and dual encirclage	5	At least 3 (unilateral)	+	+	+, hydrodissection	+, hydrodissection
			At least 6 (bilateral)				
Chang et al. 2008 [10]	Hooked pin method	5	1 (unilateral)	+	+	+, hydrodissection	+, hydrodissection
			2 (bilateral)				
Chang et al. 2009 [11]	Hooked injection needle method	5	1 (unilateral)	+	−	+, hydrodissection	+, hydrodissection
			2 (bilateral)				

SEAL: subcutaneous endoscopically assisted ligation; PIRS: percutaneous internal ring suturing.
